# Art-based beauty appreciation intervention in young adults: protocol for a two-arm active control mixed-method randomised controlled trial (ABBA-Vention)

**DOI:** 10.1186/s13063-026-09563-0

**Published:** 2026-02-25

**Authors:** MacKenzie D. Trupp, Aleksandra Igdalova, Maartje Wijnands, Blanca T. M. Spee, Eftychia Stamkou, Matthew Pelowski, Corina U. Greven

**Affiliations:** 1 Department of Medical Neuroscience, Donders Institute for Brain, Cognition and Behavior, Radboudumc, Nijmegen, The Netherlands; 2 Department of Cognition, Emotion, and Methods, Faculty of Psychology, University of Vienna, Vienna, Austria; 3 Department of Psychology, Goldsmiths, University of London, London, UK; 4 Department of Psychology, University of Amsterdam, Amsterdam, The Netherlands; 5 Karakter Child and Adolescence Psychiatry, Radboudumc, Nijmegen, The Netherlands; 6 Department of Neurology; Center of Expertise for Parkinson & Movement Disorders, Donders Institute for Brain, Cognition and Behavior, Radboud University Medical Center, Nijmegen, The Netherlands; 7 Social, Genetic and Developmental Psychiatry, King’s College London, Institute of Psychology, Psychiatry and Neuroscience, London, United Kingdom; 8 Positive Psychology Center, University of Pennsylvania, Philadelphia, PA USA

**Keywords:** Arts-based interventions, Beauty appreciation, Well-being, Burnout, Mental health, Young adults, Sensory processing sensitivity

## Abstract

**Background:**

Young adults are experiencing rising levels of mental health concerns and low well-being, which is exacerbated for around 20% of the population who are high in sensory processing sensitivity (SPS), a distinguishable, partly heritable trait associated with poorer mental health. However, despite this worrying increase in mental health issues among young adults, individuals who score highly on beauty appreciation tend to enjoy better well-being and improved mental and physical health. Several studies have shown that beauty appreciation can be taught and may serve as a cost-effective and enjoyable intervention strategy that can be easily implemented.

**Methods:**

This study has two main aims: (1) to evaluate the effectiveness of an art-based beauty appreciation intervention on enhancing beauty appreciation (primary outcome), and (2) to determine whether increasing beauty appreciation has a causal effect on reducing psychological distress and increasing mental well-being (secondary outcomes) when compared to an active matched control condition designed to isolate beauty appreciation skill development. To assess this, we will conduct a mixed-method randomised controlled trial (RCT) across three measurement points: enrollment, post-intervention, and a 4-week follow-up. *N* = 114 young adults, including those high in SPS, will be blinded and randomly allocated to the intervention or control group (~ *N* = 57/group). Our primary hypothesis is that the intervention condition will exhibit higher levels of beauty appreciation compared to the control group at the post-test while accounting for baseline differences. Our secondary hypotheses address mental well-being and psychological distress. A follow-up analysis will assess if individuals with high SPS can especially benefit and qualitative analysis will address mechanisms, barriers and facilitators between intervention and control, as well as in a high SPS subgroup.

**Discussion:**

The study aims to provide evidence that beauty appreciation skills specifically can lead to increased well-being and decreased psychological distress by including an active matched control condition that isolates this skill development. Based on positive results, the evidence will support the implementation of such interventions for young adults and highly sensitive individuals, which would be widely accessible and easy to incorporate into day-to-day life.

**Trial registration:**

ClinicalTrials.gov Registry Number: NCT06788496 (2024-12-22).

## Administrative information

Note: the numbers in curly brackets in this protocol refer to SPIRIT checklist item numbers. The order of the items has been modified to group similar items (see http://www.equator-network.org/reporting-guidelines/spirit-2013-statement-defining-standard-protocol-items-for-clinical-trials/).

**Table:** 

Title {1}	Art-based beauty appreciation intervention in young adults: Protocol for a two-arm active control mixed-method randomised controlled trial (ABBA-Vention)
Trial registration {2a and 2b}	ClinicalTrials.gov Registry Number: NCT06788496
Protocol version {3}	Version 2
Funding {4}	Trupp is supported by a Marietta Blau Mobility Fellowship funded by the Austrian Federal Ministry Republic of Education, Science and Research (BMBWF) and University of Vienna Seed Grants from the Student Office and the Vienna Doctoral School for Cognition, Behavior and Neuroscience (VDS CoBeNe). C. U. Greven is supported by an Aspasia grant from the Netherlands Organization for Scientific Research (NWO, grant number 015.015.070).
Author details {5a}	^#^MacKenzie D. Trupp, Radboudumc, Donders Institute for Brain, Cognition and Behavior, Nijmegen, the Netherlands, and University of Vienna, Faculty of Psychology, Department of Cognition, Emotion and Methods, Vienna, Austria Aleksandra Igdalova, Goldsmiths, University of London, Department of Psychology, London, UK, Positive Psychology Center, University of Pennsylvania, Philadelphia, PA, USA Maartje Wijnands, Radboudumc, Donders Institute for Brain, Cognition and Behavior Blanca T.M. Spee, Radboudumc, Donders Institute for Brain, Cognition and Behavior, Department of Neurology; University of Vienna, Faculty of Psychology, Department of Cognition, Emotion and Methods, Vienna, Austria ^+^Eftychia Stamkou, University of Amsterdam, Department of Psychology, Amsterdam, The Netherlands ^+^Matthew Pelowski, University of Vienna, Faculty of Psychology, Department of Cognition, Emotion and Methods, Vienna, Austria Corina U. Greven, Radboudumc, Donders Institute for Brain, Cognition and Behavior; Karakter Child and Adolescence Psychiatry, Nijmegen, the Netherlands, King’s College London, Institute of Psychology, Psychiatry and Neuroscience, Social, Genetic and Developmental Psychiatry, London, United Kingdom ^#^Corresponding Author ^+^Contributed Equally
Name and contact information for the trial sponsor {5b}	Radboud University Medical Center, + 31 (0) 24 361 42 44, Secretariaat.cns@radboudumc.nl
Role of sponsor {5c}	The sponsor has no say in the design or interpretation of the study.

## Introduction

### Background and rationale {6a}

The industrialised world is increasingly stimulating for young adults. Bright 24-h lights, constant traffic noise, strong influences from social media, and an inundation of conflict in the news are now the norm rather than the exception. Although humans have adapted to this increasingly overstimulating world, individuals also vary. Young people, and especially university-age students, are at the brunt of the ever-overstimulating world, with higher levels of mental health issues than ever seen before. A recent study of more than 70,000 Dutch people found that more than half of those aged 16 to 25 have mental health concerns [1].

These effects are further increased for approximately 20% of young adults due to naturally amplified sensitivity to sensory input. This group of “highly sensitive people” is described in the scientific literature as being high on the trait Sensory Processing Sensitivity (SPS). This trait is characterised by increased depth of processing, emotional reactivity and empathy, awareness of the environment's subtleties, and ease of overstimulation [2]. SPS is considered partially distinct from other personality traits [3], is moderately heritable [4], and correlates with broad functional and structural brain networks supporting sensory, cognitive, and emotional processes [5]. Young adults with high scores on SPS appear to be even more at risk for psychological issues [6] with small to medium effect sizes [7, 8]. For example, in a recent Dutch survey, people with high SPS had a three times higher burnout rate than the general population [9]. Furthermore, young adults with heightened sensitivity face an elevated vulnerability to developing mental health concerns [10–12] and report more physical health complaints [13, 14], likely due to overstimulation and lack of emotional regulation skills [11, 12, 14].

One potential avenue that could support young adults’ well-being, including highly sensitive young adults, is the practice of beauty appreciation. Large-scale cross-sectional studies suggest that trait appreciation of beauty may be a protective factor associated with well-being [15–19]. As reviewed by Trupp et al. [20], individuals with a higher appreciation of beauty, aesthetic sensitivity, or art-savouring, or a tendency to experience awe exhibit elevated subjective well-being [17, 21], greater life satisfaction [16], reduced depression [22], and improved physical health [15]. Lee et al. [15] also demonstrated predictive validity after accounting for other personality traits, like openness to experience. Notably, aesthetic sensitivity, representing an awareness and appreciation of the environment, is a fundamental part of SPS and those who are more sensitive experience more beauty and awe from the world around them [23]. Many studies suggest that of the facets of the Highly Sensitive Person Scale used to measure SPS [18], aesthetic sensitivity tends to be associated with positive health and well-being outcomes much more than other facets [24]. The past evidence indicates that cultivating an appreciation for beauty could be beneficial for young adults generally, and possibly especially so for young adults with heightened sensitivity.

In addition to studying beauty appreciation as a trait, it can also be studied as a skill. Beyond the above associational data, several experimental studies indicate that one’s tendency to appreciate their environment can also be increased [25–30]. These studies use multi-week paradigms that involve increasing exposure to beauty, teaching awareness of beauty, and reflecting on the worth of beauty. Across these interventions, evaluations of the programmes suggest positive effects. First, compared to baseline or control conditions, significant increases were found in engagement with beauty with large effects [25, 27], and appreciation of beauty [28, 29] and noticing beauty with medium effects [26]. For well-being outcomes, Martinez-Marti et al. [28] found that 97% of the sample qualitatively reported a well-being increase, while Martinez-Marti et al. [29] found significant increases in well-being compared to the control groups with moderate to large effects in a randomised control trial (RCT). However, another study [26] did not see improvements in depression or anxiety between a beauty walk group and a control walk group, possibly due to the small sample size, or more likely due to the single activity used to practice beauty repeatedly.

The link between appreciation of beauty and higher well-being in both the general population of young adults and those high in SPS, as well as previous studies showing the possibility of teaching this skill, raises the question of whether enhancing the propensity to perceive the environment as beautiful could improve well-being and mental health in—and if those high in SPS might particularly benefit from such an intervention.

### Objectives {7}

This study has two main aims: (1) to evaluate the effectiveness of a novel art-based beauty appreciation intervention (ABBA) on increasing beauty appreciation and (2) to determine if increasing beauty appreciation has a causal effect on decreasing psychological distress and increasing mental well-being by comparing these outcomes against a matched control condition which is designed to isolate beauty appreciation skill improvement to only the intervention.

Our intervention goes beyond previous studies by aggregating diverse elements from successful interventions and presenting them in an art-based learning paradigm. Utilising the arts as a tool in education has been widely successful in teaching observation skills and awareness of subtleties in medical education [31]. This study builds upon previous research through a new delivery method, aiming to create an intervention that not only fosters beauty appreciation, but also utilises cultural assets such as artworks and museums as venues to practise beauty appreciation—an activity recognised for its contributions to overall health and well-being [32].

### Main hypothesis

H1: The intervention condition will report larger increases in the primary outcome, appreciation of beauty, compared to the control group at the post-test.

H2: The intervention condition will report a larger decrease in the secondary outcome, psychological distress and a larger increase in mental well-being, compared to the control group at the post-test.

H3: The intervention condition will report higher levels of the primary outcome, appreciation of beauty, secondary outcomes: mental well-being, alongside lower levels of psychological distress compared to the control group at follow-up.

Exploratory H4: Sensory processing sensitivity will moderate improvements in both the primary and secondary outcomes.

### Trial design {8}

To evaluate this, we will conduct a mixed-method randomised controlled trial (RCT) with an active control condition assessing the impact of the ABBA intervention on trait appreciation of beauty as a primary outcome and well-being and psychological distress as a secondary outcome. The trial followed a superiority framework. There are three measurement points for the primary and secondary outcomes, including (1) enrollment, (2) a post-test after the intervention/control programme, and (3) a 4-week follow-up measure. *N* = 114 participants will be randomised into either the intervention or control group (~ *N* = 57/group). In addition, to investigate perceived mechanisms, barriers, and facilitators, participants will be selected using purposive sampling and invited to a semi-structured interview until data saturation is reached in both the intervention and control groups, with an additional subgroup analysis on those high in SPS from the intervention group. This qualitative subgroup analysis, combined with a quantitative moderation analysis, will determine whether the intervention is especially effective for those high in SPS.

## Methods: participants, interventions and outcomes

### Study setting {9}

The study is conducted via correspondence (communication through email, Microsoft Teams, online surveys and postal mail, and online-based intervention programme) in the Netherlands.

### Eligibility criteria {10}

1. Participants must be between 18–28 years of age
2. Indicate that they have a smartphone to support data collection and programme components
3. Have the ability and interest to make a trip to a museum or new location (i.e. new part of the city)
4. Be willing to complete short activities every day for two weeks
5. Be fluent in English
6. Be based in Amsterdam
7. Participants must not currently be in clinical treatment for a mental health disorder, assessed via self-report
8. Indicate that they are not currently experiencing severe psychological symptoms (i.e. depression, suicidal ideation, severe anxiety) via self-report

### Who will take informed consent? {26a}

Participants meeting the inclusion criteria will be invited to participate via email. With this invitation email, they will receive the detailed study information letter and the informed consent sheet and will be invited to schedule an online enrollment meeting via Microsoft Teams. At the enrollment meeting, the researcher will verbally explain the study. This will include the schedule and activities as well as the timeline for online surveys and the optional interview. The researcher will give the participants a chance to ask questions, and then, if the participants are ready, they will digitally sign the informed consent sheet. A follow-up meeting will be scheduled if the participant needs more time to consider joining the study. The informed consent and info sheet provide information about the storage and protection of the data and the right to leave the study at any time. The time-stamped digital informed consent will be kept according to the GDPR art.6.1.a.

### Additional consent provisions for collection and use of participant data and biological specimens {26b}

No biological specimens will be collected.

## Interventions

### Explanation for the choice of comparators {6b}

The intervention and control conditions are described in detail in Table 1, including the intervention goals, activity descriptions, and duration. Both programmes are designed to be progressive curriculums that build upon each day. All intervention programme components were selected based on previous research and have been pilot-tested. The objectives of the intervention programme (see also Table 1) are to help individuals develop an aesthetic mindset, practice observation and appreciation skills, apply these skills to their day-to-day lives, practice emotion regulation with beauty, and incorporate more beauty into their lives. The programme aims to facilitate a gradual transfer of skill from appreciation in art contexts to natural beauty and everyday life. On the other hand, the control programme is designed to match the intervention programme in all aspects except for teaching beauty appreciation and increasing exposure to art and beauty. Past studies [15, 16, 18] have not included control over increased observation skills as a possible confounding factor; instead, other studies have used active control conditions but not matched conditions that truly isolate beauty appreciation as a learned skill or behaviour.

**Table 1:** Intervention and control condition programme descriptions

	**Intervention**		**Control**	**Time (mins/day)**
	**Goal**	**Activity description**	**Equivalent activities**	
**Day 1**	Develop Aesthetic Mindset Practice Aes. Mindset and observation skills	4-minute priming video showing a landscape painting that focuses on the brushstrokes, composition, and colour Museum visit with audio guide and reflection about visit. Audio guide asking participants to slow-look at 1–3 artworks and reflect on the meaning and beauty	Priming video about learning to pay attention and being observant in everyday life Visit to be a tourist in their own city or a new city with an audio guide with directions to focus on counting things like pedestrians, types of buildings, details	120
**Day 2,3,4**	Beauty journal about artistic beauty	Written reflection on 3 experiences of beauty that they have encountered that day that is man-made	Journal listing 3 things observed that day	5–10
**Day 5**	Learning to transfer Aes. Mindset to everyday	Art photography videos focusing on slow and aesthetic looking Beauty Walk	Video of how to focus attention on everyday social interactions and learn about noticing small details Walk to practice observation	20–30
**Day 6,7,8**	Beauty journal	Taking photos or videos of natural beauty they passed during the day	Daily photo journal of descriptive photos of their observations and day life	5–10
**Day 9**	Learning about emotion regulation with beauty	Reading a short text about how beauty can induce positive emotions and with the challenge of reflecting on how beauty has impacted emotions	Reading a text about the importance of observation skills in life, focusing on emotions with the challenge to pay attention to own emotions and aspects of environment that might trigger them	5–10
**Day 10,11,** **12,13**	Practicing emotion regulation with beauty	Daily reflection on how beauty impacted emotions/stress that day. Challenge to stop, find and appreciate beauty when stressed or upset	Daily journal of things that happened that day and how that impacts emotions. Challenge to pay attention to emotions during the day	5–10
**Last day**	Develop beauty seeking behaviour	Making a written plan to add beauty to life. Like taking a trip to encounter beauty or adding beauty to the home	Making a written plan to add something new to their life to continue to practice observation skills	15

### Development of the intervention and control programmes

To prepare for this study, we designed, produced, and validated the intervention procedure. First, we conducted a literature review on past arts and beauty appreciation interventions to glean activity components and learning goals that should be incorporated into our new arts-based beauty appreciation programme. Six studies were identified, finding evidence that appreciation of beauty can be enhanced, that informed our intervention design [25–30]. These studies use multi-week programmes that involve activities aligning with four learning outcomes, including increasing exposure to beauty, learning to notice beauty, cultivating an aesthetic mindset, and reflecting on the worth or utility of beauty. In addressing these aspects, past programmes increased exposure to beauty through, for example, nature walks [25, 26] or adding plants to the house [28, 29]. To increase awareness of beauty—that is, noticing the beauty that is already part of one’s environment—directed attention tasks were used like keeping beauty journals or shooting photos of beauty to increase the habit of being aware of the beauty around them [30]. Past studies included didactic components to cultivate an aesthetic mindset, which focuses on noticing the beauty in objects in addition to their pragmatic purpose [33]. This included learning about how to appreciate mundane objects as beautiful through practice in the classroom-based sessions, for example [27]. Lastly, understanding the worth of beauty was encouraged through reflection exercises about the emotions felt during beautiful experiences [28, 29]. This latter learning goal is linked to how beauty can regulate emotions. However, the authors did not label it as such. Emotion regulation through beauty is a likely mechanism of how beauty appreciation as a skill or trait can impact well-being and mental health outcomes—known as sensory emotion regulation [34]. Having an aesthetic mindset and engaging with beauty have been shown to reduce the impact of distressing images [35], induce positive emotions [36], activate reward networks in the brain (For review see [37]), and is theorised to support distancing and emotional reappraisal [38]—key aspects of emotion regulation [39]. Thus, we developed our intervention programme activities to target these learning goals, as seen in column two of Table 1. The control programme, on the other hand, was designed to mirror the activity components but not activate these learning goals.

After designing the initial intervention and matched control programmes, we conducted a small-scale qualitative study to establish the credibility, feasibility, and comparability of the intervention and control programmes (*N* = 6, 3/condition). Participants completed a condensed version of one of the programmes, which included only one repetition of each activity (see Intervention Description Section). They were asked about feasibility issues, that is, facilitators and barriers to participation, and the effects of the intervention or skills learned in an in-depth semi-structured interview. After informed consent, online interviews were recorded on Microsoft Teams and automatically transcribed and manually checked. Feasibility issues mentioned by participants were addressed in a revision of the programmes, which included adding daily reminders, more explanatory text, and reducing the duration to two weeks instead of the planned three weeks.

The transcripts were coded based on the Braun and Clark six-step method [40] to assess learning outcomes and effects and grouped into themes. Table 2 illustrates the key themes of the intervention and control programmes’ effects or skills developed, notably revealing that feedback regarding the beauty awareness programme centred on appreciating beauty in everyday life, which was previously unnoticed. In contrast, the control programme focused on observation skills and recognising beauty in only picturesque locations. From this pilot study, we concluded that the programmes were comparable in intensity, credibility, and enjoyment and that the control condition did not enhance the appreciation of beauty in mundane spaces and objects.

**Table 2:** Comparison of the intervention and control programme effects and learning outcomes

Programme	Theme	Description
Intervention	Art seeking and appreciation	Having a desire to seek out art experiences and appreciate art more deeply and slowly
	Mood boost	Improved mood, motivated and inspired positive behaviour and thoughts
	Noticing mundane beauty	Increased awareness and appreciation of everyday objects and locations and looking beyond obvious sources of beauty
Control	Observation skills/mindfulness	Increased attention towards the environment
	Noticing beauty only in beautiful places	When in a beautiful location, noticing more details in the environment and appreciating them but not noticing more beauty in mundane objects/locations

### Intervention description {11a}

Participants will first visit an art museum (with a free entrance ticket) that they can choose from a pre-selected list of five art museums, with an accompanying audio guide developed as part of the project and instructional video. This guide and video instruct participants to view artworks slowly and to practice their observation and appreciation skills (often called “slow-looking” in museum studies [41]). From there, participants will journal three beautiful things they have encountered that day. Next, participants will be shown video art demonstrating how to observe beauty in small details (such as lentils boiling in water or waving grass), which they will be instructed to practice on a beauty walk. Following this, participants will be encouraged to take photos or videos of beautiful things they see around them in the photographer’s style (zoomed in to almost abstraction). Finally, information about regulating emotions through sensory experiences will be provided with the instruction to practice seeking beauty when stressed or having negative emotions. The information will be a short text about how our sensory experiences, such as appreciating beauty, can help to regulate emotions, summarising the latest research findings on sensory emotion regulation [34]. Finally, participants will be instructed to plan to incorporate more beauty into their lives in a self-made list (i.e. museum trips, taking a more beautiful way home, adding plants to a workspace, choosing seats with lovely views, or going for nature walks).

### Active control description

The active, matched control group will focus on developing pragmatic observation skills that do not focus on cultivating beauty appreciation. Instead, participants will be instructed to pay attention to and identify objects and elements in their surroundings without emphasizing their aesthetic value. For example, they will be asked to pay attention and count objects of the same colour they encounter, notice social behaviour and the people they pass on the street, or the types of buildings and elements in the city. The programme starts with a two-hour tourist trip to their own or a new city. They will receive a video and an audio guide that will guide them in closely observing their environment. Following this, participants will be asked to write a journal about three things they observed that day. Next, they will be shown a video demonstrating how to notice tiny details and improve observation skills, take a wider perspective, and keep track of patterns, following which they can practice on an observation walk.

Participants will practice documenting observations with their cameras. They will then be given a text about how important being observant is, especially being aware of emotions and be asked to report on their daily experiences and emotions. Here, participants will journal about their day and how daily events impact their feelings. This aims to match the intervention aspect of learning to be more aware of emotions. Finally, participants will be asked to make a written list of things they want to add to their lives to continue to practice their observation skills, like visiting a new city, or changing their routine.

### Criteria for discontinuing or modifying allocated interventions {11b}

Allocation groups will not be modified after randomisation.

### Strategies to improve adherence to interventions {11c}

Reminders are sent to participants' mobile phones before and during the two-week programme. Frequent monitoring of participant progress and adherence will be conducted, and participants not completing the programme will be contacted personally by the researcher over email to determine the reason for non-adherence and offer technical support and encouragement if needed.

### Relevant concomitant care permitted or prohibited during the trial {11d}

There are no restrictions on either group, except that the control group is asked not to visit museums or shopping malls (as a cover) during the tourist trip.

### Provisions for post-trial care {30}

There is no provision for post-trial care.

### Outcomes {12}

#### Primary outcome

The primary outcome is beauty appreciation, which will be taken at time points 1, 2, and 3, measured with the Engagement with Beauty Scale Revised (EBS-R) [42, 43], containing 18-item self-report items designed to assess cognitive and emotional engagement with natural beauty, artistic beauty, moral beauty, and beautiful ideas. Participants respond on a 7-point Likert scale, ranging from 1 (very unlike me) to 7 (very much like me). Example items include reflections on physiological responses to natural beauty, the spiritual experience elicited by art, and the desire for personal growth through moral beauty appreciation. Subscales measure engagement with moral beauty (scores 6–42) and the other three domains (scores 4–28), contributing to a total score ranging from 18 to 126. The EBS-R has demonstrated strong internal consistency and test-retest reliability (0.72 after 4 weeks), while its main strength lies in assessing trait appreciation of beauty without including other aesthetic traits, states, or emotions [44].

### Secondary outcomes

Secondary outcomes will be assessed at time points 1, 2, and 3, including mental well-being and psychological distress. Mental well-being will be measured using the Mental Health Continuum Short Form (MHC-SF) [45]. This has 14 items over three subscales: Emotional Well-being, Social Well-being (eudemonic well-being), and Psychological Well-being. The items are scored on a 6-point Likert scale (0–5), then sum-scored.

The Depression, Anxiety, and Stress Scale (DASS-21) [46] will be used to measure psychological distress. The DASS-21 has 21 items divided over three subscales, including Depression, Anxiety, and Stress. The items are assessed using a 4-point Likert scale (0–3), summed together, and then multiplied by two. This results in 3 subscales as well as a total score.

### Tertiary outcomes

Furthermore, we included tertiary outcomes, including emotion regulation, beauty awareness, attention regulation, mindfulness skills, and burnout. All Tertiary outcomes will be taken at time points 1, 2 and 3, except beauty awareness, which will be taken at time points 2 and 3. The Emotion Regulation Strategies for Artistic Creative Activities Scale (ERS-ACA; [47]) will be used with a slight modification to the question stem. The 18-item scale was designed to measure what type of emotion regulation strategies creative activities can facilitate by responding to each item in the context of “when I am engaging in [name creative activity] … I can/feel, it gives/helps/makes me, it boosts/redirects/reaffirms …” on a 5-point Likert scale from strongly disagree to strongly agree. Items can be summed into avoidance strategies (i.e., I can block out unwanted thoughts or feelings), approach strategies (i.e., it makes me reflect on my emotions), and self-development strategies (i.e., it reaffirms my identity). The stem "When I am appreciating beauty…” is used.

In addition, based on Diessner et al. [26], beauty awareness is measured by the question, “Compared to two weeks ago, I notice beauty… 1) much less than before, 2) less than before, 3) the same, 4) more than before, 5) a lot more than before”. Attention regulation will be assessed using the 20-item Attention Control Scale (ACS) [48]. The questionnaire consists of 2 subscales: (1) Attention Focusing and (2) Attention Shifting. The items are assessed on a four-point Likert scale (1-4), and items will be sum-scored. Mindfulness will be assessed using the Five Facet Mindfulness Questionnaire Short Form (FFMQ-15), with 15 items [49], with five facets: (1) Observing, (2) Describing, (3) Acting with awareness, (4) Non-judging, and (5) Non-reactivity [50]. The items are assessed on a five-point Likert scale (1-5) from rarely to always. Scores will be summed for each facet and the total score. Burnout will be measured using the Burnout Assessment Tool–12 (BAT-12), consisting of 12 items [51, 52]. This questionnaire measures burnout with four subscales: (1) Exhaustion, (2) Mental Distance, (3) Emotional Impairment, and (4) Cognitive Impairment [52]. The items are assessed on a five-point Likert scale (1-5), summed to a total score.

### Demographics and individual differences

Information about demographics and individual differences will also be collected at time point 1. This includes age, biological sex (man, female, inter-sex, prefer not to say), gender (man, woman, non-binary/non-conforming, trans-gender, prefer not to say), nationality, and education. High sensitivity will be measured using the English translation of the validated Dutch short version of the newly developed Sensory Processing Sensitivity Questionnaire – Short Form (SPSQ-SF), consisting of 26 items (SPSQ-SF) [13]. A total score can be formed in addition to six subscales: (1) Sensory Sensitivity to Subtle Internal and External Stimuli, (2) Sensory Comfort, (3) Social-Affective Sensitivity, (4) Aesthetic Sensitivity, (5) Emotional and Physiological Reactivity, and (6) Sensory Discomfort. The first four subscales load onto a positive SPS dimension, whereas the last two load onto a negative SPS dimension [53]. The items are assessed on a seven-point Likert scale (1-7), sum scored together.

Furthermore, two bespoke questions will be asked to evaluate participants’ past experience with the arts, including interest and engagement: “How interested are you in art, music, or visiting art/cultural spaces? (1 not at all, 7, very much)”; second, “In the past year, how often have you engaged with/attended/visited arts venues (including music events, the theatre/dance, concerts, art museums, art festivals, exhibitions, either in-person or online, etc.)? 1) More than once a month, 2) once a month, 3) once every two or three months, 4) three times per year, 5) twice per year, 6) once a year, 7) not in the past year.” These two questions will then be summed together and treated as a continuous variable. These questions are based on the art interest construct from the widely used Vienna Art Interest and Knowledge scale [54]. Finally, openness to experience will be measured with the openness facet of the Big Five Inventory (BFI; [55]), which has ten questions. These questions are measured on a 1 to 5 Likert scale (strongly agree to strongly disagree) and will be summed together.

### Expectation, credibility, adherence, and enjoyment

To understand how participants’ expectations and adherence might impact the results of the trial, several aspects will be assessed at time point 1. Expectation and credibility will be measured through the Credibility and Expectancy Questionnaire (CEQ), which has six questions about how much participants expect the programme to benefit their well-being [56]. We have slightly modified this questionnaire from its intended use as a clinical instrument for therapies, asking about improvement in functioning by replacing the term “functioning” with “well-being” in the questions.

To measure adherence, as part of the programme, each day, participants will also be asked to indicate whether they fully, partially or did not complete the programme elements for that day. These questions were introduced in the enrollment meeting with emphasis on the need for honesty that would not impact any compensation. Adherence will also be monitored via the number of sign-ins and their progress in the online programmes. Finally, enjoyment of the programmes will be assessed on a 1–7 Likert scale.

### Qualitative interview

A semi-structured qualitative interview after timepoint 3 will be conducted with a subset of participants focusing on the effects and learning outcomes of the intervention and control programmes, mechanisms, barriers and facilitators. We will follow the Consolidated Criteria for Reporting Qualitative Research (COREQ; [57]).

### Participant timeline {13}

#### Enrollment and baseline

In an enrollment meeting hosted on Microsoft Teams, the researcher will walk the participant through the selected programme components and instructions with a PowerPoint presentation and provide time for questions. As listed in the information sheet, advertisement, and screening survey, the participants will be asked for their postal address so that the login details for the respective programme and the free museum tickets (intervention only) can be sent via post. Participants will set up an account on the Samply Research Application [58] to receive reminders about the study. At the end of the meeting, the researcher will explain that the surveys will be sent to them via email (via Castor). Once the participant has no further questions, the enrollment meeting will finish. The participants will then be sent the baseline survey. The list of baseline questions is provided in the overview in Table 3.

**Table 3:** Outcomes and timepoints of assessment

Assessment tools	Baseline T1	Post T2	Follow-up T3
**Demographics and covariates**			
Demographics (6 items)	x		
SPSQ-SF (26 items)	x		
Experience with Arts (bespoke; 2 items)	x		
BFI-O (10 items)	x		
**Primary outcome**			
EBS-R (18 items)	x	x	x
**Secondary outcomes**			
MHC-SF (14 items)	x	x	x
DASS-21 (21 items)	x	x	x
**Tertiary outcomes**			
ERS-ACA (18 items)	x	x	x
Beauty Awareness Question (1 item)		x	x
FFMQ-15 (15 items)	x	x	x
ACS-12 (12 items)	x	x	x
BAT-12 (12 items)	x	x	x
Interview*			x
**Additional questions**			
CEQ (6 items)	x		
Adherence and Enjoyment (2 items)		x	
Additional background questions (2 items)			x

*SPSQ-26 *Sensory Processing Sensitivity, *BFI-O* Big Five Inventory-Openness, *EBS-R* Engagement with Beauty Revised, *MHC-SF* Mental Health Continuum Short Form, *DASS-21* Depression, Anxiety, and Stress Scale-21 item, *ERS-ACA* Emotion Regulation Strategies for Artistic Creative Activities Scale, *FFMQ-15* Five Facets Mindfulness Questionnaire Short Form, *ACS-12* Attention Control Scale-12 item, *BAT-12* Burnout Assessment Tool-12 item, *CEQ* Credibility and Expectancy Questionnaire. *Interviews will only be conducted in a subsample (*n* = 25) until data saturation

### Intervention and control programmes

After finishing the baseline survey and the research materials arrive by post, the participant can access the online programme platform (hosted on Thinkific, an online course-sharing platform) to start the programme. The programme is followed daily for 14 days by logging into Thinkific on their personal phones or computers (see Intervention section for intervention procedure description).

### Post-survey, follow-up survey and compensation

The post-survey will be sent once participants have completed the 14-day programme. The list of surveys is in Table 3. At this time, participants are reminded that in 4 weeks, they will be sent the final survey and, following its completion, their compensation. Once the participant has completed it, as confirmed by checking the ID codes of the completed surveys, they will be awarded their Dutch online shopping voucher (Bol.com) and research credits.

### Semi-structured qualitative Interviews

After follow-up (4 weeks post-intervention), participants selected through purposive sampling (selecting to maximise diversity by varying levels of programme completion, gender, age, and level of SPS) will be sent an invitation to participate in a qualitative interview with the information sheet and the interview-specific informed consent sheet. The interviews will be conducted one-on-one by one of the study’s researchers on Microsoft Teams, which allows recording and automatic transcription. Transcripts will be sent to interviewees to check all is correct. Participants will be compensated with an online shopping voucher. The interview description can be found in the Outcomes Section.

### Sample size {14}

**Fig. 1:**
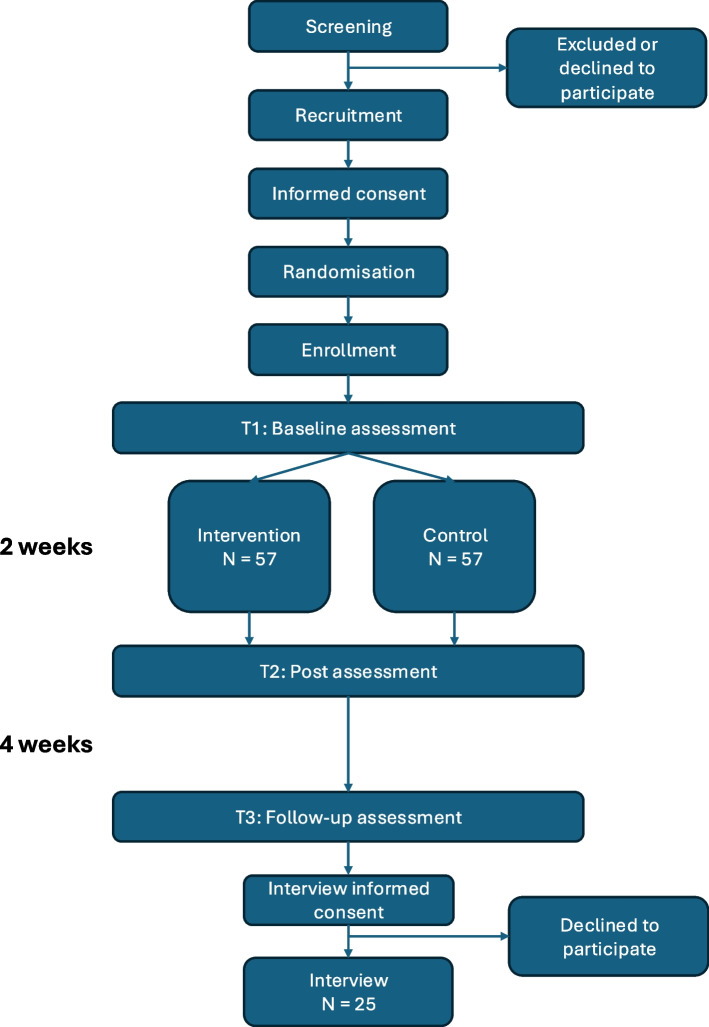
Participant timeline

The power analysis is based on the primary hypothesis that appreciation of beauty will be higher post-test in the intervention group than in the control group. Effect sizes for appreciation of beauty range from *d* = 0.27 to *d* = 0.58 in previous RCTs [27, 29]. Due to our active control group, we based our power calculation on a small to moderate effect size of *d* = 0.35 for an ANCOVA where the baseline is the covariate. Power analyses were run using the software G*power and based on the procedure by [59]. This method obtains the N per group from a t-test multiplied by a design factor (D). The design factor is *D* = 1-*r*^2^, where *r* is the pre-and post-test correlation. We base *r* on the four-week test-retest reliability of the Experience of Beauty Scale of 0.72 [44]; thus, *D* = 1-0.72.72.72.72.72^2^ =. 48. Based on the power of 80% for a two-tailed test with an alpha of 0.05 and an estimated effect size of 0.35, 102*0.48 = 49/group are needed. Approximately 15% are added for the estimated dropout rate, bringing the total sample size to *N* = 114. See Fig. 1.

For the qualitative interviews, as is common in qualitative research, it is not yet possible to determine how many participants will participate in the interviews. Participants will continue to be approached and interviewed until the interviews no longer generate new information (i.e. data saturation is reached). The point at which saturation occurs depends, among other factors, on the heterogeneity of the sample. Given that our sample is relatively homogeneous (mostly university students and young adults in Amsterdam), we anticipate that a sample of approximately *N* = 20 participants will ultimately be included, *N* = 10 per group. An additional *N* = 5 high SPS individuals from the intervention group will be selected for a subgroup analysis. If participants do not accept the invitation, we will contact replacements from the remaining pool of intervention condition participants.

### Recruitment {15}

Participants will be recruited through the participant platform at the University of Amsterdam and via social media. The participant platform advertises studies to students and provides them with research credits they must complete as part of their undergraduate curriculum. An advertisement will be placed on the participant platform that includes necessary study information with a link for interested participants to click to fill out a screening survey (via online survey platform Qualtrics, https://www.qualtrics.com) and provide their contact email. This survey starts with the information letter and a screening informed consent. The intervention and control programmes will be advertised as targeting observation skills while being a tourist in your city. There is no mention of art or beauty appreciation in the advert. In addition, advertisements will be placed on social media with links to the study information and the same screening survey.

## Assignment of interventions: allocation

### Sequence generation {16a}

Participants will be randomised in blocks of 2 and 4 via a sequence generated by the electronic data capture system Castor (https://www.castoredc.com).

### Concealment mechanism {16b}

The sequence for randomisation is concealed from researchers within Castor. Further, using blocks of 2 and 4 reduces the possibility of guessing the next condition.

### Implementation {16c}

The enrollment researchers will assign participants to conditions during the enrollment meeting, after informed consent, using the automatic randomisation sequence generated by Castor.

## Assignment of interventions: blinding

### Who will be blinded {17a}

Participants are blinded to the allocated condition. Although the information sheet specifies that there are two conditions, it is unclear to participants which is the control and which is the intervention due to the high-quality, credible active control condition. It is not possible in this study to keep the researchers blind, as they are responsible for explaining the programmes during the enrollment meeting.

### Procedure for unblinding if needed {17b}

After the study, participants will be unblinded as to which condition they were allocated and offered a full explanation of the study design, aims, and a description of both conditions. This will be done in writing, with the additional offer to discuss the study at the participant’s request. At this time, participants will also be provided information about existing mental health services and support.

## Data collection and management

### Plans for assessment and collection of outcomes {18a}

The collection of outcome data will be done via surveys and semi-structured interviews. Below are detailed explanations for assessment and collection plans for each stage of the study, as well as procedures for all participant contact. The researchers collecting data will be trained PhD candidates and master’s level researchers. All survey data will be collected via the Castor Electronic Data Capture system. All interviews will be conducted and recorded on Microsoft Teams.

### Plans to promote participant retention and complete follow-up {18b}

Participants are informed of their right to leave the study at any time without consequences to themselves. Reasons for withdrawals and timing will be documented. To promote participant retention, we will send reminders to those who stop having poor adherence and ask all withdrawn participants to complete the post and follow-up tests where possible. Participants who withdraw are not replaced if they have completed the pre-survey.

### Data management {19}

The research team will frequently monitor the completion of survey data for completeness and send reminders to participants to amend incomplete data. The interview will be audio recorded, automatically transcribed using Microsoft Teams, and manually edited to remove identifying information. Transcripts will be sent to participants for approval (member checks). The audio file will be destroyed after the transcription has been approved.

### Confidentiality {27}

This study will be conducted according to the Declaration of Helsinki version 2013 principles and following the Good Clinical Practice (GCP) guidelines promulgated by the International Conference on Harmonization (ICH). The handling of personal data will comply with the General Data Protection Regulation 2018 (GDPR), in Dutch, the “Algemene verordening gegevensbescherming” (AVG).

The researcher will give each participant a unique identification code. Participant contact details and linking identification codes will be saved in a password-protected file and stored separately from the data. The data will be stored following RadboudUMC protocols. The analysis, when necessary for data protection, will be conducted in a secure Digital Research Environment (DRE). Data will not be published; however, further researchers can request access after signing a data-access agreement. The principal investigator will destroy documents containing personal information, such as consent forms or non-anonymised data, after 15 years.

### Plans for collection, laboratory evaluation and storage of biological specimens for genetic or molecular analysis in this trial/future use {33}

No biological specimens will be collected.

## Statistical methods

### Statistical methods for primary and secondary outcomes {20a}

The main analysis will be conducted in two stages. The first is according to the intention-to-treat (ITT) analysis principle [60], with all participants included where possible. This includes dropouts and non-completers of the programmes. Second, we will repeat the analysis Per-Protocol (PP) [60] to gain a clearer picture of the intervention-related effects, excluding non-completers or poor-quality data (see exclusions below). To evaluate whether there are differences between the conditions at the post-test, considering baseline differences, a series of ANCOVAs with the condition as the IV and the post-test as the DV, with baseline scores, sex, and age as covariates will be run on primary and secondary outcomes. To evaluate the long-term effects of the intervention and the control condition, linear mixed models (LMM) will be fitted to predict each outcome variable. The same approach will be applied to evaluate the tertiary outcomes.

For more details, see our pre-registration: osf.io/q8c6u.

### Interim analyses {21b}

No Interim analyses will be done.

### Methods for additional analyses (e.g. subgroup analyses) {20b}

To understand the pattern of an individual response, we will calculate the Reliable Change Index (RCI) for pre-post change scores, expecting more individuals in the intervention condition to reliably improve on primary, secondary, and tertiary outcomes than in the control condition [61]. Furthermore, factors including adherence, expectancy and credibility, past art experience and personality will be compared between groups. If the groups differ significantly on any scale, we will include that scale as an exploratory covariate.

Finally, we will assess any differential effect of the intervention on highly sensitive individuals in three ways. First, we expect SPS to predict a greater likelihood of improvement in the RCI analyses. Second, moderation analysis will be used to assess whether individuals with higher scores on SPS experience larger increases in primary and secondary outcomes from the intervention. Third, qualitative sub-group analysis will be used to investigate mechanisms, barriers and facilitators for a high SPS subgroup, by comparing their responses to a group of low and moderate SPS individuals.

### Methods in analysis to handle protocol non-adherence and any statistical methods to handle missing data {20c}

In ITT analysis, we will only exclude participants who do not have pre- and post-data for the DV in question. We will record reasons for dropout and look at baseline measures between groups to assess the randomness of missing data. If data are found to be random, missing outcome data will be used as the last observation carried forward approach [62]. In PP analysis, participants will be excluded if they a) fail two out of three of the attention of the checks (1 per survey), b) signed into the programme less than seven times or c) self-reported not completing more than seven activities, indicating they have done less than half of the programme (for more details see the pre-registration).

The qualitative data will be analysed using thematic analysis within the qualitative software package Atlas.ti (version 24.1.1), employing a constant comparison method and adhering to the framework of Braun and Clarke [40], utilising open coding [63]. Two researchers will code the transcripts (a PhD candidate and an MSc level researcher). The first five transcripts will be independently coded by both coders and subsequently compared, while the remaining transcripts will be coded by one researcher and checked by the other. Mapping the codes involves examining the transcript, coding, grouping codes into themes and subthemes, and identifying the core concept through axial and selective coding [63, 64], moving back and forth between the codes and the original data [64]. The coders will meet regularly to discuss the codes and emerging patterns, as well as to assess when data saturation has been achieved.

### Plans to give access to the full protocol, participant level-data and statistical code {31c}

Pre-registration: osf.io/q8c6u.

## Oversight and monitoring

### Composition of the coordinating centre and trial steering committee {5d}

The coordinating centre comprises the principal investigator, the lead researcher, and other personnel. The principal investigator oversees the scope of the experiment, while the lead researcher manages the daily operations and research team, including recruitment, enrollment, data collection, monitoring, analysis and reporting. Weekly meetings occur between the lead researcher and the research personnel and bi-weekly meetings are held between the entire team to track progress. Emergency meetings are convened as necessary. There is no involvement from a steering committee or stakeholders.

### Composition of the data monitoring committee, its role and reporting structure {21a}

There is no monitoring committee; the research team conducts the monitoring.

### Adverse event reporting and harms {22}

No adverse events or harms are expected from this study.

### Frequency and plans for auditing trial conduct {23}

There are no plans for conducting an audit. However, an audit can be conducted randomly as part of RadboudUMC protocols.

### Plans for communicating important protocol amendments to relevant parties (e.g. trial participants, ethical committees) {25}

Major amendments will be submitted to the ethical committee.

### Dissemination plans {31a}

The study results will be published in academic journals, at scientific conferences, and in reports and social media posts for lay audiences and stakeholders.

## Discussion

In order to reduce the high levels of poor mental health among young adults generally, and especially those who are highly sensitive who are at increased risk, this study aims to determine whether an art-based intervention can enhance beauty appreciation and, consequently, improve mental well-being while alleviating psychological distress. This addresses the need for enjoyable, non-pharmacological interventions that leverage leisure to support health and well-being [65].

Previous research has predominantly examined the relationship between beauty appreciation—also known as aesthetic sensitivity—and mental and physical health outcomes through cross-sectional studies [15–19], often implying causality without experimental validation. Several studies have evaluated interventions aimed at increasing beauty appreciation skills; however, earlier intervention studies have lacked active control conditions that isolate the specific effects of enhancing beauty appreciation from other aspects, such as heightened general observation skills or attention regulation skills. Therefore, despite the considerable potential, it remains unclear whether increasing beauty appreciation in young adults is a suitable candidate for developing a large-scale intervention that could be implemented in universities or schools. This is also particularly relevant for highly sensitive young adults, for whom interventions are limited, and there have been calls to gain a deeper understanding of methods that can support those with high Sensory Processing Sensitivity (SPS).

Overall, this study possesses several strengths. First, we developed the intervention alongside a rigorously designed active control condition, validated through a qualitative pilot study. This enabled us to address feasibility issues and confirm that the control condition primarily enhances observation skills rather than beauty appreciation. Methodologically, this high-quality control condition supports blinding measures and minimises expectancy effects by ensuring a credible and engaging condition. This also supported reducing participant self-selection effects and minimising expectancy or placebo effects, as we were able to promote the study as a general observation intervention, where neither beauty appreciation, the arts, nor museums were mentioned.

Beyond these aspects, the mixed-method design facilitates a more comprehensive assessment of outcomes and underlying mechanisms, providing deeper insights into the intervention’s effectiveness and mechanisms and allowing for a more nuanced understanding of how highly sensitive individuals might experience the intervention programme and its effects. Finally, by implementing the study in a real-world setting, we enhance the ecological validity of the findings, offering a clearer understanding of how the intervention may function outside a controlled trial environment. This approach strengthens the study’s potential for practical application and broader impact, aligning with the lifestyle or community-based intervention strategy that seeks to utilise behaviours and leisure activities to support health and well-being in addition to, or at times in place of, more clinical interventions.

## Trial status

Recruitment started in November 2024 and will continue until June 2025.

## Data Availability

Data will be available upon request and data agreement with the RadboudUMC from the corresponding author.

## Abbreviations

SPS: Sensory processing sensitivity
RCT: Randomised controlled trial
ABBA: Arts-based beauty appreciation
D: Design factor
EBS-R: Engagement with Beauty Scale Revised
MHC-SF: Mental Health Continuum Short Form
DASS-21: Depression, Anxiety, and Stress Scale- 21 item
ERS-ACA: Emotion Regulation Strategies for Artistic Creative Activities Scale
ACS: Attention Control Scale
FFMQ-15: Five Facets Mindfulness Questionnaire Short Form
BAT-12: Burnout Assessment Tool-12 item
SPSQ-SF: Sensory Processing Sensitivity Questionnaire—Short Form
BFI-O: Big Five Inventory-Openness
CEQ: Credibility and Expectancy Questionnaire
ITT: Intention-to-treat
PP: Per-protocol
LMM: Linear mixed models
COREQ: Consolidated Criteria for Reporting Qualitative Research
RCI: Reliable Change Index
GCP: Good Clinical Practice
ICH: International Conference on Harmonization
GDPR: General Data Protection Regulation
AVG: Algemene verordening gegevensbescherming
DRE: Digital Research Environment
